# Genes encoding conserved hypothetical proteins localized in the conjugative transfer region of plasmid pRet42a from *Rhizobium etli* CFN42 participate in modulating transfer and affect conjugation from different donors

**DOI:** 10.3389/fmicb.2014.00793

**Published:** 2015-01-14

**Authors:** Eunice López-Fuentes, Gonzalo Torres-Tejerizo, Laura Cervantes, Susana Brom

**Affiliations:** Programa de Ingeniería Genómica, Centro de Ciencias Genómicas, Universidad Nacional Autónoma de MéxicoCuernavaca, Mexico

**Keywords:** quorum-sensing, conjugation, transcriptional regulation, hypothetical proteins, rhizobia

## Abstract

Among sequenced genomes, it is common to find a high proportion of genes encoding proteins that cannot be assigned a known function. In bacterial genomes, genes related to a similar function are often located in contiguous regions. The presence of genes encoding conserved hypothetical proteins (chp) in such a region may suggest that they are related to that particular function. Plasmid pRet42a from *Rhizobium etli* CFN42 is a conjugative plasmid containing a segment of approximately 30 Kb encoding genes involved in conjugative transfer. In addition to genes responsible for Dtr (DNA transfer and replication), Mpf (Mating pair formation) and regulation, it has two chp-encoding genes (RHE_PA00163 and RHE_PA00164) and a transcriptional regulator (RHE_PA00165). RHE_PA00163 encodes an uncharacterized protein conserved in bacteria that presents a COG4634 conserved domain, and RHE_PA00164 encodes an uncharacterized conserved protein with a DUF433 domain of unknown function. RHE_PA00165 presents a HTH_XRE domain, characteristic of DNA-binding proteins belonging to the xenobiotic response element family of transcriptional regulators. Interestingly, genes similar to these are also present in transfer regions of plasmids from other bacteria. To determine if these genes participate in conjugative transfer, we mutagenized them and analyzed their conjugative phenotype. A mutant in RHE_PA00163 showed a slight (10 times) but reproducible increase in transfer frequency from *Rhizobium* donors, while mutants in RHE_PA00164 and RHE_PA00165 lost their ability to transfer the plasmid from some *Agrobacterium* donors. Our results indicate that the chp-encoding genes located among conjugation genes are indeed related to this function. However, the participation of RHE_PA00164 and RHE_PA00165 is only revealed under very specific circumstances, and is not perceived when the plasmid is transferred from the original host. RHE_PA00163 seems to be a fine-tuning modulator for conjugative transfer.

## Introduction

The availability of sequenced genomes has increased exponentially in the last years. At present, there are 168 complete sequences of archaea, 2788 of bacteria, and 222 of eukarya, according to NCBI. Analyses of the sequences usually show the presence of genes similar to others with known functions, but, invariably, genes with unknown function are present. Some of them are “orphans” found only in a specific strain, while others are shared among various organisms, encoding conserved hypothetical proteins (chp).

Bacteria belonging to the rhizobia are able to form nitrogen-fixing symbiosis with the roots of leguminous plants (Masson-Boivin et al., 2009). The genomes of these bacteria are usually composed of a chromosome and various plasmids of sizes ranging between 150 and 1800 Kb. The plasmids may carry up to 40% of the total genomic content, including the information allowing the establishment of the symbiosis (Romero and Brom, 2004). Also, some of the plasmids have been shown to carry genes involved in other bacterial functions, such as LPS biosynthesis (García de los Santos and Brom, 1997), metabolic functions (Villaseñor et al., 2011) vitamin synthesis, and even some functions essential for bacterial maintenance (Landeta et al., 2011). A characteristic commonly ascribed to plasmids is the ability to perform conjugative transfer (CT). The elements required for CT are a set of genes involved in the processing of DNA (Dtr, DNA transfer and replication), a set of genes involved in formation of the mating pair (Mpf, Mating pair formation), and an *oriT* site, where transfer is initiated (de la Cruz et al., 2010). Various rhizobial plasmids have been shown to have this capacity. They have been grouped according to their transfer genes into four types (Ding and Hynes, 2009; Giusti et al., 2012) those regulated by: (I) quorum-sensing, (II) the RctA-repressor, (III) those lacking a Mpf system, and (IV) those containing other regulators. Accordingly, these plasmids contain segments with the Dtr, Mpf, *oriT* and regulatory genes. Additionally, some of them also contain genes encoding conserved hypothetical proteins. As these chp-encoding genes are intercalated between transfer related genes, we hypothesized that they may be involved in this function. To analyze this, we studied the participation of the chp- encoding genes localized in the transfer region, in the CT ability of plasmid pRet42a of *Rhizobium etli* strain CFN42.

## Materials and methods

### Bacterial strains and plasmids

The bacterial strains and plasmids used in this work are described in Supplementary Table 1. *Rhizobium* and *Agrobacterium* strains were grown on PY medium (peptone-yeast extract medium supplemented with CaCl_2_ at a final concentration of 4.5 mM) at 30°C (Noel et al., 1984). *Escherichia coli* strains were grown in LB medium (Miller, 1972), at 37°C. When required, antibiotics were added at the following concentrations: nalidixic acid, 20 μg/ml; kanamycin, 15 or 30 μg/ml; gentamicin, 30 μg/ml; rifampin, 50 or 100 μg/ml; erythromycin, 25 μg/ml; spectinomycin, 100 μg/ml; neomycin, 60 μg/ml; streptomycin, 100 μg/ml; and tetracycline, 2 μg/ml.

### Bacterial matings

Conjugation between *E. coli* and *R. etli* was done biparentally, using *E. coli* S17-1 (Simon, 1984) as the donor. Transconjugants were selected with the appropriate antibiotics. Conjugation experiments were performed on PY plates at 30°C, using overnight cultures grown to stationary phase. Donors and recipients were mixed in a 1:2 ratio and incubated overnight. The mixtures were collected and suspended in 1 ml of 10 mM MgSO4–0.01% (vol/vol) Tween 40. Serial dilutions were plated on suitable selective media. The transfer frequency was expressed as the number of transconjugants per donor cell.

### PCR

All oligonucleotides used (Table 1) were synthesized at the Unidad de Síntesis Química IBT-UNAM. PCR amplification was carried out with Taq polymerase (Invitrogen). PCR conditions consisted of 30 cycles of 94°C for 1 min, 56–64°C for 1 min and 72°C for 1 min.

**Table 1:** **Oligonucleotides used in this study**.

**Gene**	**Sequence**	**Position**	**PCR product**
RHE_PA00163^a^	F: ^5′^GCTGAATTCCACGGCCACGATTGCTT^3′^ R: ^5′^CAGGGATCCCGCCATCGAGGATCACT^3′^	176724–176740 176461–176477	332 bp
RHE_PA00164^a^	F: ^5′^TGCGAATTCCCGCAAACGCTTGTTCGA^3′^ R:^5′^ACGGGATCCCCTCGACGATTTCCGCTGT^3′^	177208–177226 176967–176985	316 bp
RHE_PA00165^a^	F: ^5′^AAAGAATTCACAAGCCGATGCTCTCT^3′^ R: ^5′^CAGGGATCCTACCACATCGATGCTCG^3′^	177735–177751 177577–177593	175 bp
Complete RHE_PA00163^b^	F:^5′^AAGCTGCAGTCCGTGAAGCGCCTGAGC ^3′^ R:^5′^AGGGGATCCCGTTGGATCGGCAGAAAT^3′^	176329 –176346 176817–176834	506 bp
Complete RHE_PA00163, RHE_PA00164 and RHE_PA00165^c^	F:^5′^GATGAATTCACTGAAAGCGTCGAGAAAGGC ^3′^ R:^5′^GTCGGATCCGAGGAGCCGACGGTGTTCCCG^3′^	176294–176323 178063–178092	1798 bp

a *These products were used to construct the mutants. The F oligonucleotides contained a PstI site, and the reverse contained a BamHI. The introduced bases are underlined*.

b *This product was used to clone the complete RHE_PA00163, a PstI site was introduced in the F oligonucleotide, and a BamHI restriction site in the R. The introduced bases are underlined*.

c *This product was used to clone the complete RHE_PA00163, RHE_PA00164 and RHE_PA00165. An EcoRI site was introduced in the F oligonucleotide, and a BamHI restriction site in the R. The introduced bases are underlined*.

### Construction of mutant derivatives

RHE_PA00163 and RHE_PA00164 mutants were constructed by interrupting the genes with pK18*mob* (Schäfer et al., 1994) introduced by recombination. RHE_PA00163 was mutagenized with plasmid pK18*mob*-*163* (pK18*mob* with a 332 bp *Eco*RI-*Bam*HI internal fragment of RHE_PA00163). Recombination creates two incomplete copies of the gene. One of them lacks 41 bp of the 3′ end, while the other lacks 57 bp of the 5′ end. RHE_PA00164 was mutagenized with plasmid pK18 *mob*-*164* (pK18*mob* with a 326 bp *Eco*RI-*Bam*HI fragment of RHE_PA00164). One of the copies lacks 173 bp of the 3′ end, ending at nucleotide position 459, while the other lacks 200 bp of 5′ end.

To construct a mutant in RHE_PA00165, an internal fragment was cloned with *Eco*RI-*Bam*HI in the pK18-*mob-sacB* suicide vector (Schäfer et al., 1994), using the molecular techniques from Sambrook et al. (1989). The *sacB* gene confers lethal susceptibility to sucrose, allowing for positive selection of double recombinants. The pK18-*mob- sacB*-*165* plasmid was digested with *Eco*RV, and a Sp cassette was introduced in this site, generating pK18-*mob-sacB*-165::Sp. This plasmid was used to obtain a mutant in RHE_PA00165 by double recombination, selecting for spectinomycin-resistant, sucrose-resistant colonies. All the constructs were checked by PCR.

### Cloning of the wild-type genes

The pTE3-*163* plasmid, containing the entire RHE_PA00163, was constructed by cloning a 506 bp fragment (generated with *Taq* polymerase High Fidelity (Invitrogen) and engineered to contain the appropriate cloning sites), into the *Pst*I/*Bam*HI in the multiple cloning site of the vector pTE3 (Egelhoff and Long, 1985), which contains a strong constitutive promoter.

Plasmid pWR, containing the three hcp genes RHE_PA00163, RHE_PA00164 and RHE_PA00165 was constructed by cloning a 1798 bp fragment, containing their own promoters, into the *EcoR*I/*Bam*HI sites of pBBR1MCS-5 (Kovach et al., 1995).

### Measurement of β-glucuronidase activity

Cultures of *R. etli* derivatives harboring transcriptional fusions were grown to stationary phase. Quantitative *uidA* activity was measured in 1 ml culture samples with p-nitrophenyl glucoronide as a substrate, as described by Girard et al. (2000).

### Bioinformatics analyses

For the construction of the XRE phylogenetic trees, the proteins were aligned with the module of Clustal implemented in MEGA5 (Tamura et al., 2011). The models of protein evolution for our sequences were selected with ProtTest 2.4 (Abascal et al., 2005). The model selected was LG +I+G. Maximum likelihood (ML) trees were inferred under the selected model using PhyML v3.1 (Guindon and Gascuel, 2003). The robustness of the ML topologies was evaluated by bootstrap analysis implemented in PhyML v3.1 (100 replicates). We employed the best of NNIs and SPRs algorithms to search the tree topology and 100 random trees as initial trees. The accession numbers are indicated in the figure.

BLASTP analysis on the NCBI and https://img.jgi.doe.gov servers were used to get homologs and examine the neighborhood of the selected genes.

## Results

### The transfer region of pRet42a contains conserved hypothetical genes and an XRE-type regulator

**Figure 1:**
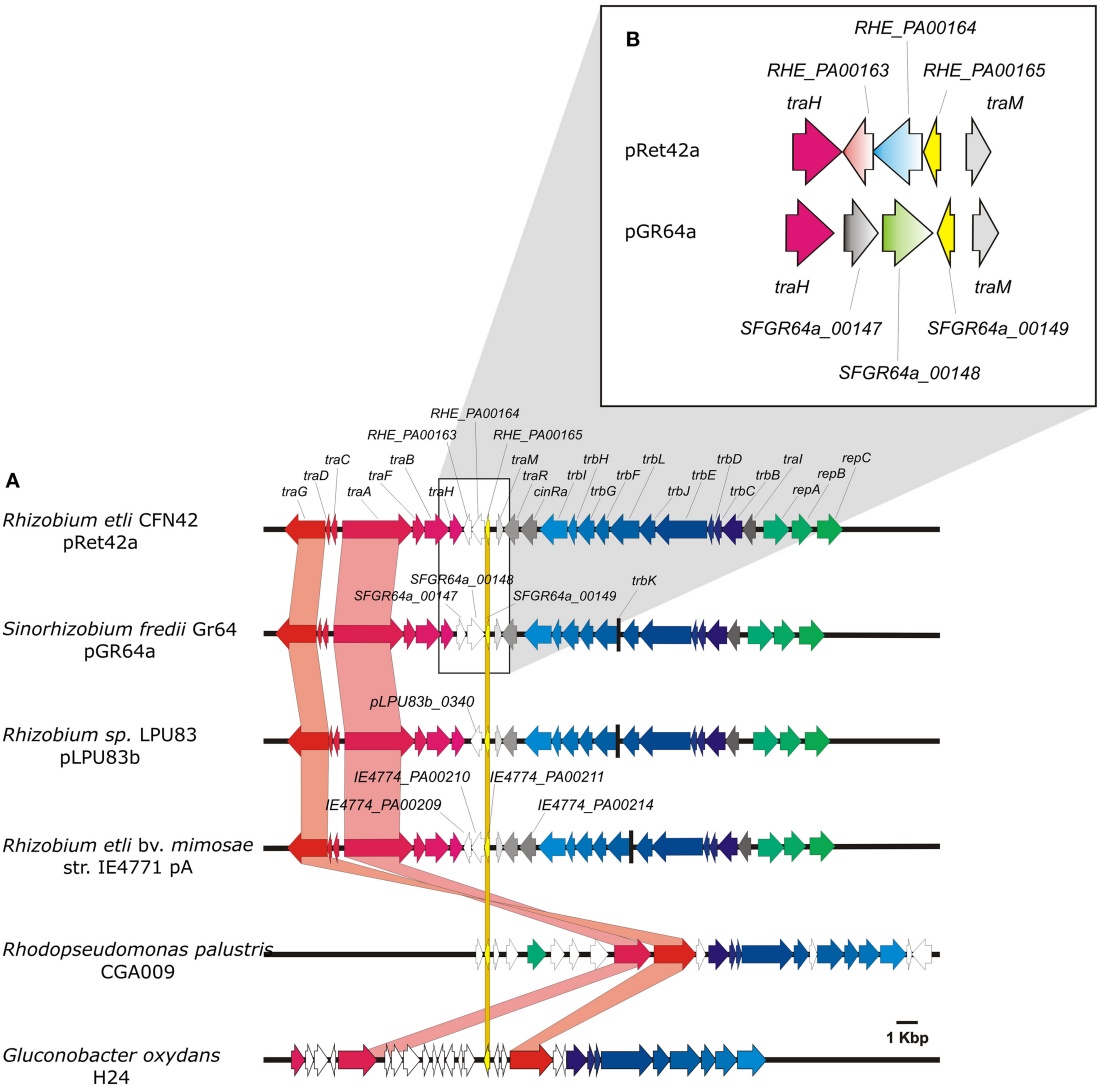
**Organization of the genetic regions localized next to XRE regulators**. **(A)** Comparison of the transfer regions, including the Dtr and Mpf genes. Orthologs are marked with the same color. Important traits are highlighted. Green, Blue, and Red-tones indicate Replication, Mpf and Dtr genes, respectively. Gray arrows indicate regulatory genes and empty arrows hypothetical protein encoding genes. **(B)** Zoom-in showing the organization of genes located between *traH* and *traM* of pRet42a and pSfr64a.

*Rhizobium etli* strain CFN42 (Quinto et al., 1982) contains 6 plasmids, named pRet42a to pRet42f, ranging in size from 185 to 650 Kb. Plasmid pRet42a is a conjugative plasmid, whose transfer genes are regulated by quorum-sensing (Tun-Garrido et al., 2003). Plasmid pRet42d corresponds to the symbiotic plasmid (pSym) this plasmid is able to perform conjugative transfer through cointegration with pRet42a (Brom et al., 2004). As previously mentioned, rhizobial plasmids have been classified in four groups; among them pRet42a belongs to group I, and pRet42d to group II. This classification is supported by the phylogenetic analyses of the relaxase (*traA*) gene (Ding and Hynes, 2009; Giusti et al., 2012). Recently, we described that group I could be split into three sub-groups, in this classification group I-B harbors pRet42a (Torres Tejerizo et al., 2014). Phylogenetic analyses of *traA* and *traR* showed that the genes from pRet42a are very closely related to those of pSfr64a, a conjugative plasmid from *Sinorhizobium fredii* GR64 (Cervantes et al., 2011). Here, we compared the Dtr and Mpf regions of these plasmids (Figure 1A), and found that, although they are mostly similar, they present a few remarkable differences. The similarities include the general organization, with the Dtr genes localized in two divergent operons next to the *oriT* site, and the Mpf genes located adjacent to the replication genes, also in a divergent operon, where the first gene of the operon is the regulatory *traI* gene. One difference was that while pRet42a encodes a *cinR* regulator (Tun-Garrido et al., 2003) pSfr64a does not. Also, both plasmids present three unclassified genes between the last Dtr gene (*traH*) and *traM*. pRet42a harbored RHE_PA00163, RHE_PA00164 and RHE_PA00165; pSfr64a had SFGR64a_00147, SFGR64a_00148, and SFGR64a_00149. By means of BLAST analysis on the NCBI and https://img.jgi.doe.gov servers, we detected that all these genes are conserved hypothetical proteins present in several bacteria. Three of them contain conserved domains of unknown function: RHE_PA00163 presents a COG4634 domain, RHE_PA00164 has a DUF433 domain, and SFGR64a_00148 presents a DUF1814. Proteins RHE_PA00165 and SFGR64a_00149 are predicted as transcriptional regulators with conserved domains, belonging to the Helix-turn-helix XRE-family like proteins. These prokaryotic DNA binding proteins are described as proteins that respond to xenobiotic elements. Only SFGR64a_00147 showed no conserved domains, but we have determined that it is required for efficient conjugative transfer of plasmid pSfr64a (unpublished results).

Regarding the organization of the genes, RHE_PA00163, RHE_PA00164, and RHE_PA00165, as well as SFGR64a_00149 are transcribed divergent to *traH*, while SFGR64a_00147 and SFGR64a_00148 are encoded in the same direction as *traH* gene (Figure 1B).

The fact that hypothetical genes are present in the transfer regions of the two plasmids shown above, led us to question if the similar genes present in other bacteria are also localized next to transfer regions.

### Genes similar to RHE_PA00163, RHE_PA00164 and RHE_PA00165 are localized in the transfer regions from plasmids present in diverse organisms

**Figure 2:**
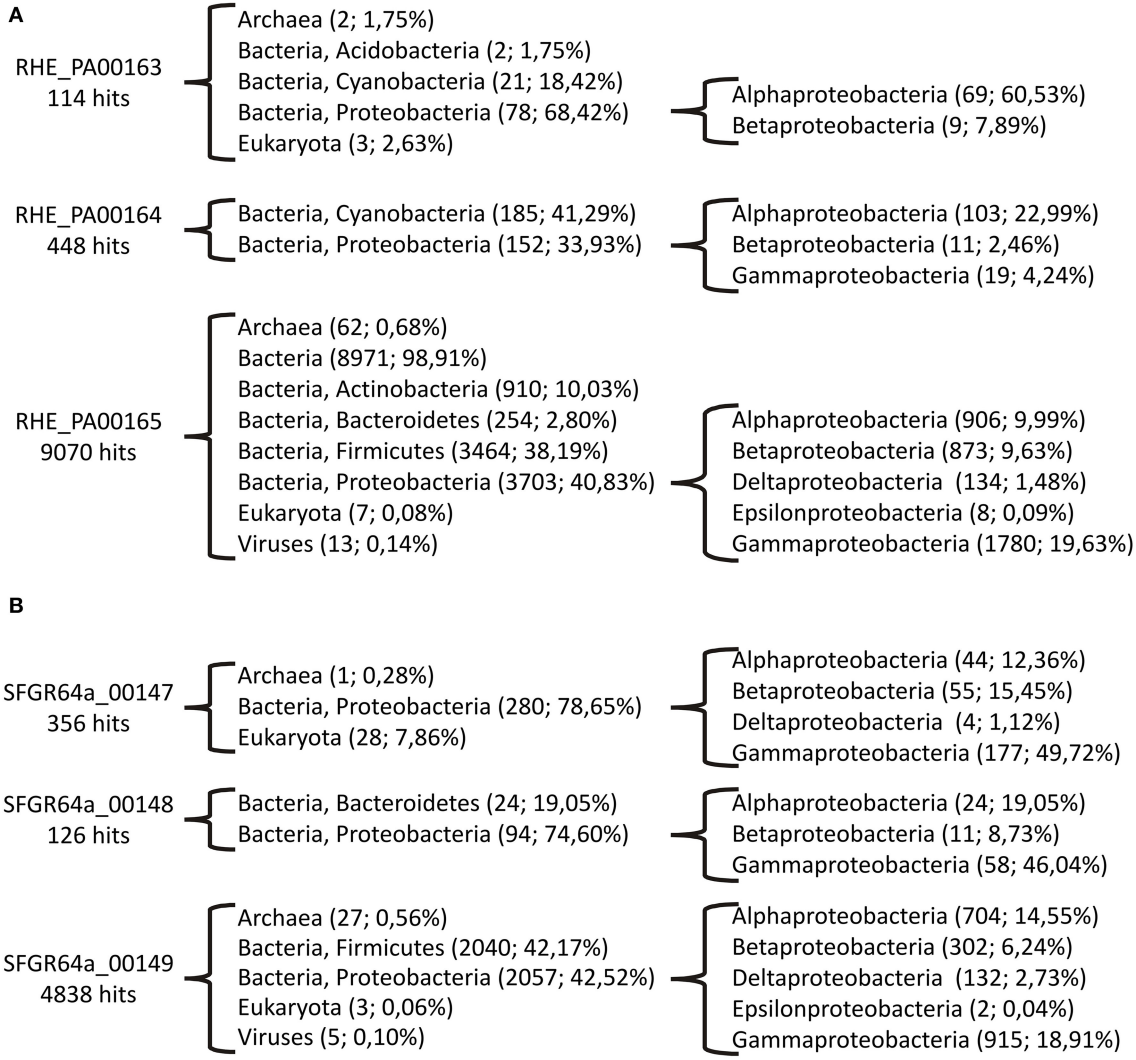
**Organisms that present homologs to hypothetical orfs**. **(A)** From *R. etli* CFN42. **(B)** From *S. fredii* GR64. Homologs were searched for by BLASTP, The number of hits and the percent over the total hits are shown in parenthesis.

To determine the range of organisms showing similar gene clusters, we analyzed the distribution and diversity of homologs of the hypothetical proteins. Initially we performed a BLASTP analysis for each protein against the nr database. A high number of matches were found, and thus a minimum of 30% of identity was set to reduce the number of hits. This percentage of identity is the usually accepted cut-off to define orthologs (Rost, 1999). With this threshold, we obtained 114, 448 and 9070 hits for *R. etli* hypothetical proteins RHE_PA00163, RHE_PA00164, and RHE_PA00165, respectively; and 356, 126 and 4838 hits for *S. fredii* hypothetical proteins SFGR64a_00147, SFGR64a_00148, and SFGR64a_00149 (Figure 2, Supplementary Table 2).

RHE_PA00163 hits were mostly distributed in Proteobacteria (68.42% of the hits), with a few in Cyanobacteria and two hits in Archaea. RHE_PA00164 had 33.93% hits in Proteobacteria and 41.29% in Cyanobacteria. RHE_PA00165 presented the highest diversity: hits were found in Archaea, Bacteria, Eukaryota and Viruses. Among Bacteria, Firmicutes and Proteobacteria harbored most of the hits (38.19 and 40.83%, respectively), while in the Proteobacteria Phylum, Gammaproteobacteria (19.63%) carried most of the homologs.

In the case of the *S. fredii* hypothetical proteins, homologs to SFGR64a_00147 and SFGR64a_00148 were mostly present in Gammaproteobacteria (49.72 and 46.04%), with some hits in Alphaproteobacteria (12.36 and 19.05%) and Betaproteobacteria (15.45 and 8.73%), remarkably, some hits were found in Eukaryota. For SFGR64a_00149, homologs were distributed among Firmicutes and Proteobacteria, and in this Phylum, Gammaproteobacteria were the most represented (18.91%), this gene also had hits in Archaea Eukaryota.

These results display the wide distribution of the chp-encoding genes from the transfer region of pRet42a. It is probable that horizontal gene transfer events could be related to their presence in very diverse organisms, including Archaea, Virus and Eucaryota.

**Figure 3:**
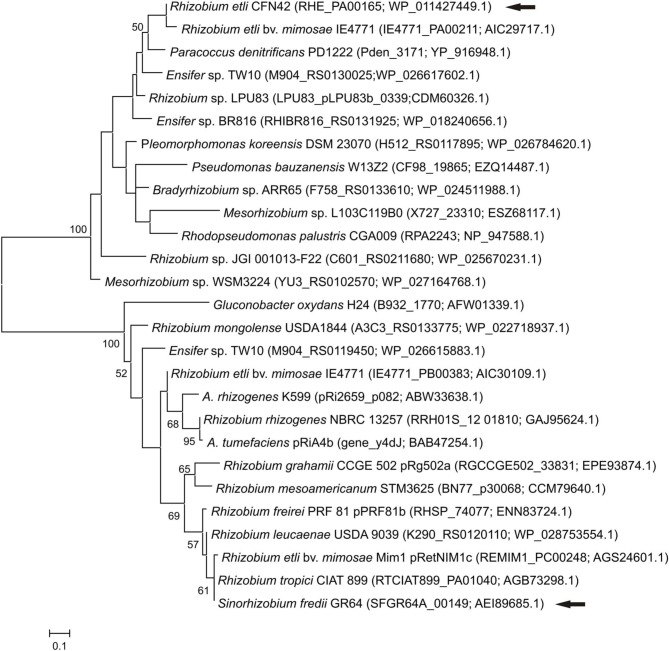
**Phylogenetic relationship of XRE-type regulators**. The phylogenetic tree was based on the XRE-type regulators. Analyses were conducted by means of the Maximum Likelihood method. Arrows indicate the XRE regulators from pRet42a and pSfr64a. The locus tag and accession numbers are shown in parenthesis. Bootstrap values higher that 50 are shown at the nodes. Bar indicates substitution/site.

Even if both, SFGR64a_00149 and RHE_PA00165, possess a XRE domain and are similarly located upstream of a *traM* regulator, a phylogenetic analysis showed that they are not closely related (Figure 3).

In addition to being widely distributed in diverse genomes, these chp-encoding genes are located in the neighborhood of Dtr and Mpf gene clusters of several bacteria, such as *Rhizobium etli* bv. *mimosae* IE4771 (pA), *Ensifer* sp. TW10, *Rhizobium* sp. LPU83 (pLPU83b) (Wibberg et al., 2014), *R. leguminosarum* bv viciae 8401(pRL1JI) (Danino et al., 2003), *Rhodopseudomonas palustris* CGA009 (Larimer et al., 2004), *Rhizobium tropici* CIAT 899 (pA) (Ormeño-Orrillo et al., 2012), *Rhizobium etli* bv. *mimosae* Mim1 (pRetNIM1c), *Rhizobium leucaenae* USDA 9039, *Rhizobium freirei* PRF 81 (pPRF81b), *Rhizobium mesoamericanum* STM3625, *Rhizobium grahamii* CCGE 502 (pRg502a) (Althabegoiti et al., 2012) and *Gluconobacter oxydans* H24 (Figure 1A), the genomic island of *E. coli* Nissle 1917 (Grozdanov et al., 2004) and the symbiosis island of *Mesorhizobium loti* R7A (Ramsay et al., 2013). The orthologs of RHE_PA00163 located near transfer genes, and/or next to RHE_PA00164 orthologs are indicated in Supplementary Table 2.

The broad distribution and conserved position of these chp-encoding proteins hints that they may participate in the conjugative transfer phenomena.

### The hypothetical genes from pRet42a affect conjugative transfer of the plasmid

In order to determine if the chp-encoding genes localized in pRet42a participate in conjugative transfer, we constructed mutant derivatives, and analyzed their phenotype. RHE_PA00163 and RHE_PA00164 were interrupted with pK18mob, and RHE_PA00165 by a spectinomycin resistance cassette, as described in Materials and Methods.

#### Mutation of RHE_PA00163 increases CT frequency in wild-type background

The derivative carrying a mutation in RHE_PA00163 showed a 10-fold increase in conjugative transfer frequency compared to the wild type strain (Table 2). Interestingly, this phenotype was only observed when the donor carried all the other endogenous plasmids of the strain. It was not observed when the donor lacked the symbiotic plasmid pRet42d, or pRet42f, or from an *Agrobacterium* donor (data not shown). This suggests that the modulation effect caused by RHE_PA00163 may involve elements localized in these plasmids. Also, the transfer frequency varies with different recipient strains; compare lines 1 vs. 3, and 2 vs. 4 in Table 2. We see that transfer frequency is higher when UIA143 (Farrand et al., 1989) is used as recipient. However, the increase in transfer frequency of the mutant in RHE_PA00163 compared to the wild type is maintained, indicating that, in addition to the effect of the mutation on the transfer frequency of pRet42a, there is also an effect of the recipient.

**Table 2:** **Conjugation frequencies from rhizobial donors^a^**.

**Donor**	**Characteristics**	**Transfer frequency^b^**
(1) CFNX187	Wild type, labeled pRet42a (Brom et al., 1992)	1.4, 1.6 × 10^−1^
(2) CE3-*163*::pK18mob	RHE_PA00163 mutant	3.7, 3.0 × 10^0^
(3) CFNX187	wild type, labeled pRet42a	4.1, 4.0 × 10^−3^
(4) CE3-*163*::pK18mob	RHE_PA00163 mutant	1.6, 2.0 × 10^−1^
(5) CE3-*163*::pK18mob/pTE3-*163*	RHE_PA00163 mutant complemented with cloned RHE_PA00163	1.3, 0.02 × 10^−1^
(6) CE3-*164*::pK18mob	Mutant in RHE_PA00164	1.1, 0.01 × 10^−1^
(7) CE3-*165*::Sp	Mutant in RHE_PA00165	5.27, 0.74 × 10^−1^

a *The recipient in crosses 1, 2 5, 6, and 7 was UIA143, and CFN2001 (Leemans et al., 1984) in crosses 3 and 4*.

b *Transfer frequency is expressed as number of transconjugants per donor cell, and is the average of at least three experiments*.

#### Expression levels of traI decrease in the RHE_PA00163 mutant

Previously, we determined that conjugative transfer of pRet42a depends on quorum-sensing regulation mediated by *traI, traR* and *cinR* (Tun-Garrido et al., 2003). To determine if the increase in transfer frequency of the RHE_PA00163 mutant was due to an increase in the expression level of *traI*, we introduced plasmid pCT7 (pBBMCS53/*traIp-uidA*) carrying a transcriptional fusion of *traI* (Tun-Garrido et al., 2003), into the mutant in RHE_PA00163 and determined the ß-glucuronidase activity, in comparison to the wild-type strain. Surprisingly, the results showed that the expression level of *traI* decreased in the mutant background (Table 3). This indicates that the increase in transfer frequency in the mutant depends on elements different from the TraI quorum-sensing regulator.

**Table 3:** **Expression levels of *traI***.

**Strain**	**Characteristics**	**Expression level^a^**
CE3/*traIp-uidA*	Wild type, labeled pRet42a, *traI* fusion	11.9 ± 1.19
CE3-*163*::pK18mob, *traIp-uidA*	RHE_PA00163 mutant, *traI* fusion	4.2 ± 0.65
CE3*-163*::pK18mob, *traIp-uidA*, pTE3-*163*	RHE_PA00163 mutant complemented with cloned RHE_PA00163 *traI* fusion	7.5 ± 0.81

a Expression level is expressed as ß glucuronidase specific activity (nm/min/mg of prot), it is the average of at least three experiments and the SD is shown.

#### Complementation of the RHE_PA00163 mutant with the wild-type gene

We cloned the complete RHE_PA00163 in a vector able to replicate in *Rhizobium*, as described in Material and Methods. This clone was introduced into the RHE_PA00163 mutant containing pCT7 (pBBMCS53/*traIp-uidA*). We determined the ß-glucuronidase activity and the results showed that, although it did not reach the level of the wild-type, the complemented strain partially restored the expression level of *traI* (Table 3). Also, the transfer frequency decreased in the complemented strain (Table 2). A possible explanation for this is that RHE_PA00163 has a dual role in conjugative transfer, with a positive effect on *traI* expression, and a negative effect on some unidentified participant, able to induce a fine-tuned increase in transfer.

### RHE_PA00164 and RHE_PA00165 are required for transfer from different agrobacterium donors

The derivatives lacking functional RHE_PA00164 or RHE_PA00165 showed transfer frequencies similar to those of the wild-type strain from rhizobial donors (Table 2), but a different phenotype was observed when the plasmid carrying the mutation was transferred from *Agrobacterium* donors (Table 4). The strain carrying a mutation in RHE_PA00164 was unable to generate transconjugants when the donor was *Agrobacterium* strain GMI9023 (Rosenberg and Huguet, 1984), a derivative that lacks its endogenous pTi and pAT plasmids, however, the mutant is still able to conjugate from an *Agrobacterium* donor that lacks the pTi, but conserves the pAT (strain UIA143). The plasmid with a mutation in RHE_PA00165 also lost its ability to transfer from GMI9023. Additionally, its transfer frequency from UIA143 was lower than that of the wild-type plasmid. Both mutants acquired the wild-type phenotype when a plasmid carrying the three hcp-encoding genes was introduced. This plasmid did not alter the transfer frequency of the wild-type plasmid, although it carries the whole region, possibly because, as mentioned earlier, the effect of RHE_PA00163 is not observed from *Agrobacterium* donors (Table 4). These data suggest that RHE_PA00164 and RHE_PA00165 do participate in conjugative transfer, but their activity is masked in their native background.

**Table 4:** **Conjugation frequencies from *Agrobacterium* donors^a^^,^^b^**.

**Donor**	**Characteristics**	**Transfer frequency**
GMI9023/p42a::Tn5	Plasmid-less *Agrobacterium*, labeled pRet42a	1.64 ± 0.66 × 10^−4^
GMI9023/p42a::Tn5, pWR	Plasmid-less *Agrobacterium* with labeled pRet42a and pBBR1MCS5 containing the three hcp genes	1.92 ± 1.28 × 10^−4^
UIA143/p42a::Tn5	*Agrobacterium* with pAT and labeled pRet42a	5.90 ± 2.39 × 10^−5^
UIA143/p42a::Tn5, pWR	*Agrobacterium* with pAT, the labeled pRet42a and pBBR1MCS5 containing the three hcp genes	9.60 ± 5.80 × 10^−5^
GMI9023/p42a-*164*::pK18	Plasmid-less *Agrobacterium*, pRet42a with RHE_PA00164 mutant	ND
UIA143/ p42a-*164*::pK18	*Agrobacterium* with pAT, with RHE_PA00164 mutant	1.31 ± 0.35 × 10^−5^
GMI9023/p42a-*164*::pK18, pWR	RHE_PA00164 mutant in GMI9023 with pBBR1MCS5 containing the three hcp genes	7.36 ± 2.94 × 10^−5^
UIA143/p42a-*164*::pK18, pWR	RHE_PA00164 mutant in UIA143 with pBBR1MCS5 containing the three hcp genes	7.01 ± 3.11 × 10^−5^
GMI9023/p42a*−165*::Sp	Plasmid-less *Agrobacterium*, pRet42a with RHE_PA00165 mutant	ND
UIA143/p42a*-165*::Sp	*Agrobacterium* with pAT, pRet42a with RHE_PA00165 mutant	2.9 ± 1.2 × 10^−6^
GMI9023/p42a-*165*::pK18, pWR	RHE_PA00165 mutant in GMI9023 with pBBR1MCS5 containing the three hcp genes	1.46 ± 0.59 × 10^−5^
UIA143/p42a-*165*::pK18, pWR	RHE_PA00165 mutant in UIA143 with pBBR1MCS5 containing the three hcp genes	5.5 ± 2.7 × 10^−5^

a *Transfer frequency is expressed as N° transconjugants per donor cell, and is the average of at least three experiments*.

b *The recipient in these crosses was CFN2001*.

## Discussion

The analyses presented in this paper, regarding the distribution of hypothetical protein-encoding genes and XRE-type regulators similar to those localized in the transfer region of plasmid pRet42a from *R. etli* CFN42, show that these genes are widely distributed among bacteria, and even some archaea and eukaryotic organisms (Figure 2). The highest proportion of homologs to RHE_PA00163 and RHE_PA00164 was found in Alphaproteobacteria (60.53 and 22.99% of them in rhizobiales, respectively). At least 45 of the species detected contained both, RHE_PA00163 and RHE_PA00164, usually localized close to each other. Interestingly, in plasmids of various organisms such as *Rhizobium etli* bv. *mimosae* IE4771 (pA), *Ensifer* sp. TW10, *Rhizobium* sp. LPU83 (pLPU83b), *Rhodopseudomonas palustris* CGA009, *Rhizobium tropici* CIAT899 (pA), *Rhizobium etli* bv. *mimosae* Mim1 (pRetNIM1c), *Rhizobium leucaenae* USDA9039, *Rhizobium freirei* PRF81 (pPRF81b), *Rhizobium mesoamericanum* STM3625, *Rhizobium grahamii* CCGE 502 (pRg502a) and *Gluconobacter oxydans* H24 the genes were localized next to Dtr and/or Mpf clusters, as exemplified in Figure 1A.

Regarding SFGR64a_00147 and SFGR64a_00148 from *S. fredii* GR64, they showed a similar distribution of homologs, mostly among proteobacteria, with the highest proportion present in Gammaproteobacteria (45%) many of these were found in *Escherichia* genera. Also in some of these strains, the homologs were localized in genomic islands (e.g., Nissle 1917) or plasmids.

The XRE-type regulators presented the highest number of homologs, 9070 for RHE_PA00165 and 4838 for SFGR64a_00149 (Figure 2). Although both contain an XRE-type domain, these two orfs are phylogenetically distant (Figure 3). All these data suggest that these genes may participate in the conjugative transfer of bacterial plasmids, and even of genomic islands.

The functional studies of the chp-encoding *orfs* from pRet42a showed that RHE_PA00163 participates as a fine-tuning modulator of transfer, possibly through components encoded in plasmids pRet42d and pRet42f, as donors lacking these plasmids did not show the increase in transfer frequency. Additionally, we found that a mutation in this *orf* leads to a decrease in the expression of *traI*. These results suggest that RHE_PA00163 differently affects elements involved in transfer, having a positive effect on *traI*, and a negative one on other elements, which are able to induce a slight increase in transfer in the absence of RHE_PA00163. How does RHE_PA00163 achieve its effects? It could be a directly interacting with the different elements or it could be an indirect effect. Another open question for further research is if the protein product of the gene is responsible, or if the effect is mediated through RNA.

RHE_PA00164 and RHE_PA00165 also showed a role in conjugative transfer, although in this case the effect was only revealed in conjugation from non-native *Agrobacteria* donors. It is possible that these *orfs* are only expressed in the heterologous background. The fact that the RHE_PA00164 and RHE_PA00165 mutants only were able to transfer from the donor containing plasmid pAT, suggests that the conjugative ability is probably due to their interaction with genes encoded in plasmid pAT.

Due to their organization, it would be possible that RHE_PA00163, RHE_PA00164, and RHE_PA00165 form an operon. However, our experimental data shows that mutation in each of the genes presents an independent phenotype. Also, we performed a search for putative promoters using the BPROM program for prediction of bacterial promoters (Solovyev and Salamov, 2011). The results indicate the presence of a putative promoter for each gene (Supplementary Table 3). A recent paper by López-Leal et al. (2014) shows that the transcription levels of the three genes differ greatly among them. In another paper (Vercruysse et al., 2011) it can be seen that RHE_PA00163 is regulated by (p)ppGpp, while RHE_PA00164 and RHE_PA00165 are not affected. All these data suggest that these genes are transcribed independently, and do not form an operon. However, it is still possible that under some conditions the genes could be transcribed as an operon.

Some data have begun to emerge regarding the participation in conjugative transfer of genes similar to those described in this paper. In plasmid pRleVF39b of *R. leguminosarum* bv viciae strain VF39 it was shown that a XRE-type regulator encoded close to the Dtr genes functions as a repressor of conjugative transfer (Ding et al., 2013). In the symbiosis island of *M. loti* R7A, the gene named *qseC*, encoding a XRE type regulator was shown to participate in regulation of excision and transfer of the island (Ramsay et al., 2013). In *S. meliloti* strain LPU88, plasmid pLPU88a mobilizes pLPU88b, inactivation of a hypothetical encoding protein gene localized in pLPU88a resulted in its inability to promote transfer of pLPU88b from *S. meliloti* strain 2011, but was dispensable from the native LPU88 background (Pistorio et al., 2013). In *S. fredii* GR64, we have found that a mutation in SFGR64a_00147 impairs transfer of pSfr64a (our unpublished results).

The examples from the literature, in conjunction with the data presented in this work implicate the participation of genes with unknown function localized near transfer regions in this process. Their mode of participation seems to be variable, some as positive effectors, others as repressors, some acting as modulators, and others showing an absolute requirement. Additionally, they seem to depend on interaction with elements encoded in other replicons.

## Conflict of interest statement

The authors declare that the research was conducted in the absence of any commercial or financial relationships that could be construed as a potential conflict of interest.
