# Supplementary material for: Genes encoding conserved hypothetical proteins localized in the conjugative transfer region of plasmid pRet42a from Rhizobium etli CFN42 participate in modulating transfer and affect conjugation from different donors
Source: Front Microbiol. 2015 Jan 14;5:793. doi: 10.3389/fmicb.2014.00793 (PMC4294206; doi:10.3389/fmicb.2014.00793)
Supplement: Supplementary file 1 [file Table1.DOCX]

***Supplementary Material***

**Genes encoding conserved hypothetical proteins localized in the conjugative transfer region of plasmid pRet42a from *Rhizobium etli* CFN42 participate in modulating transfer and affect conjugation from different donors.**

**Eunice López-Fuentes ^1,2^, Gonzalo Torres-Tejerizo^1,3^, Laura Cervantes ^1^, and** **Susana Brom^1*^**

^1^Programa de Ingeniería Genómica, Centro de Ciencias Genómicas, Universidad Nacional Autónoma de México, Cuernavaca, Mor. México.

^2^Present address: División de Biología Molecular, Instituto Potosino de Investigación Científica y Tecnológica. San Luis Potosí, SLP, México.

^3^Present address: Instituto de Biotecnología y Biología Molecular, UNLP, CCT-La Plata-CONICET. Departamento de Ciencias Biológicas, Facultad de Ciencias Exactas, Universidad Nacional de La Plata, La Plata, Argentina.

*** Correspondence:** Susana Brom, Programa de Ingeniería Genómica, Centro de Ciencias Genómicas, Universidad Nacional Autónoma de México, Av Universidad 1001, Cuernavaca, Mor., CP 62210, México. sbrom@ccg.unam.mx

## Suplementary Tables

**Supplementary Table 1. Strains and plasmids**

| **Strain or plasmid** | **Relevant features** | **Reference or source** |
| --- | --- | --- |
| *Rhizobium etli* |  |  |
| CFN42 | Wild type, Nal^R^ | Quinto *et al*. 1982 |
| CFN2001 | CFN42 derivative (p42a⁻ p42d^⁻^) | Leemans *et al*. 1984 |
| CFNX182 | CFN42 derivative (p42a⁻) | Brom *et al*. 1992 |
| CFNX187 | CFNX182 complemented with p42a::Tn5*mob* | Brom *et al*. 1992 |
| CFN42/pBBMCS53 | CFN42 derivative containing pBBMCS53 | This work |
| CFN42-*163*::pK18 | CFN42 derivative, RHE_PA00163::pK18*mob* | This work |
| CE3-*163::*pK18/pBBMCS53 | CFN42 derivative, RHE_PA00163::pK18*mob* with pBBMCS53 | This work |
| CE3-*163*::pK18/pCT7 | CFN42 derivative, RHE_PA00163::pK18*mob* containing pCT7(pBBMCS53/*traIp-uidA*) | This work |
| CFNX672 | CFN42 derivative containing pCT7(pBBMCS53/*traIp-uidA*) | Tun-Garrido *et al*. 2003 |
| CE3*-163*::pk18mob/pTE3-*163* | CFN42 derivative RHE_PA00163*3*::pk18mob with pTE3- RHE_PA00163 | This work |
| CE3-*164*::pK18mob | CFN42 derivative, RHE_PA00164::pK18*mob* | This work |
| CE3*-165*::Sp | CFN42 derivative, RHE_PA00165::Sp | This work |
| *Agrobacterium tumefaciens* |  |  |
| UIA143 | C58 derivative *recA*⁻ pTi⁻ | Farrand *et al*. 1989 |
| GMI 9023 | C58 cured of its native plasmids (pTi⁻ pAT⁻) | Rosenberg & Huguet. 1984 |
| UIA143/p42a::Tn5 | UIA143 derivative containing p42a::Tn5 | This work |
| UIA143/p42a-*163*::pK18 | UIA143 derivative containing p42a/ RHE_PA00163::pK18mob | This work |
| UIA143/p42a-*164*::pK18 | UIA143 derivative containing p42a/ RHE_PA00164::pK18mob | This work |
| UIA143/p42a-*165*::Sp | UIA143 derivative containing p42a/ RHE_PA00165::Sp | This work |
| UIA143/p42a-*164*::pK18, pWR | UIA143 derivative containing p42a/ RHE_PA00164::pK18mob and pBBR1MCS-5 containing the whole region with the hcp genes RHE_PA00163, RHE_PA00164 and RHE_PA00165 | This work |
| UIA143/p42a-*165*::pK18/, pWR | UIA143 derivative containing p42a/ RHE_PA00165::pK18mob and pBBR1MCS-5 containing the whole region with the hcp genes RHE_PA00163, RHE_PA00164 and RHE_PA00165 | This work |
| GMI9023/p42a::Tn5 | GMI9023 derivative containing p42a::Tn5 | This work |
| GMI9023/p42a-*163*::pK18 | GMI9023 derivative containing p42a RHE_PA00163::pK18mob | This work |
| GMI9023/p42a-*164*::pK18 | GMI9023 derivative containing p42a RHE_PA00164::pK18mob | This work |
| GMI9023/p42a-*165*::Sp | GMI9023 derivative containing p42a RHE_PA00165::Sp | This work |
| GMI9023/p42a-*164*::pK18, pWR | GMI9023 derivative containing p42a-RHE_PA00164::pK18mob and pBBR1MCS-5 containing the whole region with the hcp genes RHE_PA00163, RHE_PA00164 and RHE_PA00165 | This work |
| GMI9023/p42a-*165*::pK18, pWR | GMI9023 derivative containing p42a-RHE_PA00165::pK18mob and pBBR1MCS-5 containing the whole region with the hcp genes RHE_PA00163, RHE_PA00164 and RHE_PA00165 | This work |
| *Escherichia coli* |  |  |
| DH5α | *supE44* ∆*lacU169* (ф80*lacZ*∆M15) *hsdR17 recA1 endA1 gyrA96 thi-1 relA1* | Sambrook *et al*. 1989 |
| S17-1 | C600::RP4-2 (Tc::Mu)(Km::Tn7) | Simon, 1984 |
| S17/pCT7 | Containing pCT7 | Tun-Garrido *et al*. 2003 |
| Plasmids |  |  |
| pK18*mob* | Suicide vector, Km^R^ | Schäfer *et al*. 1994 |
| pK18*mobsacB* | Suicide vector, Km^R^ | Schäfer *et al*. 1994 |
| pBBR1MCS-5 | Cloning vector, Gm^R^ | Kovach *et al*. 1995 |
| pCT7 | pBBMCS53/*traIp-uidA* | Tun-Garrido *et al*. 2003 |
| pBBMCS53 | ∆p*lacZ*pBBRIMCS-5 containing *uidA* gene promoter of pWM5, Gm^R^ | Girard *et al.* 2000 |
| pTE3 | Cloning vector, containing *trp* promoter | Egelhoff & Long. 1985 |
| pK18*mob*-*163* | pK18*mob* with a 332 bp *Eco*RI-*Bam*HI fragment of RHE_PA00163 | This work |
| pK18*mob*-*164* | pK18*mob* with a 326 bp *Eco*RI-*Bam*HI fragment of RHE_PA00164 | This work |
| pK18-*mob-sacB*-*165*::Sp | pK18mobsacB with a 175 bp *Eco*RI-*Bam*HI fragment of RHE_PA00165 interrupted with a Sp cassette | This work |
| pTE3-*163* | pTE3 containing the entire RHE_PA00163 (514 pb *Pst*I-*Bam*HI) | This work |
| pWR | pBBR1MCS-5 containing the whole region with the hcp genes RHE_PA00163, RHE_PA00164 and RHE_PA00165 | This work |
