# Supplementary material for: Genes encoding conserved hypothetical proteins localized in the conjugative transfer region of plasmid pRet42a from Rhizobium etli CFN42 participate in modulating transfer and affect conjugation from different donors
Source: Front Microbiol. 2015 Jan 14;5:793. doi: 10.3389/fmicb.2014.00793 (PMC4294206; doi:10.3389/fmicb.2014.00793)
Supplement: Supplementary file 3 [file Table3.DOCX]

***Supplementary Material***

**Genes encoding conserved hypothetical proteins localized in the conjugative transfer region of plasmid pRet42a from *Rhizobium etli* CFN42 participate in modulating transfer and affect conjugation from different donors.**

**Eunice López-Fuentes ^1,2^, Gonzalo Torres-Tejerizo^1,3^, Laura Cervantes ^1^, and** **Susana Brom^1*^**

^1^Programa de Ingeniería Genómica, Centro de Ciencias Genómicas, Universidad Nacional Autónoma de México, Cuernavaca, Mor. México.

^2^Present address: División de Biología Molecular, Instituto Potosino de Investigación Científica y Tecnológica. San Luis Potosí, SLP, México.

^3^Present address: Instituto de Biotecnología y Biología Molecular, UNLP, CCT-La Plata-CONICET. Departamento de Ciencias Biológicas, Facultad de Ciencias Exactas, Universidad Nacional de La Plata, La Plata, Argentina.

*** Correspondence:** Susana Brom, Programa de Ingeniería Genómica, Centro de Ciencias Genómicas, Universidad Nacional Autónoma de México, Av Universidad 1001, Cuernavaca, Mor., CP 62210, México. sbrom@ccg.unam.mx

## Suplementary Tables

**Supplementary Table 3. Localization of putative promoters for RHE_PA00163,** **RHE_PA00164 and RHE_PA00165 from pRet42a (NCBI accesion CP000134.1)**.

| **Gene** | **Localization of putative promoter** |
| --- | --- |
| RHE_PA00163  complement (176398..176775) | -10: 176 834 - 176 842  -35: 176885 - 176 860 |
| RHE_PA00164  complement (176772..177404) | -10: 177 429 - 177 437  -35: 177 449 - 177 454 |
| RHE_PA00165  complement (177508..177729) | -10: 177 858 - 177 866  -35: 177 881 - 177 886 |
